# Inoculation works and health advocacy backfires: Building resistance to COVID-19 vaccine misinformation in a low political trust context

**DOI:** 10.3389/fpsyg.2022.976091

**Published:** 2022-10-25

**Authors:** Li Crystal Jiang, Mengru Sun, Tsz Hang Chu, Stella C. Chia

**Affiliations:** ^1^Department of Media and Communication, City University of Hong Kong, Hong Kong, Hong Kong SAR, China; ^2^College of Media and International Culture, Zhejiang University, Hangzhou, China

**Keywords:** vaccine, inoculation, vaccines, message resistance, health promotion, Hong Kong

## Abstract

This study examines the effectiveness of the inoculation strategy in countering vaccine-related misinformation among Hong Kong college students. A three-phase between-subject experiment (*n* = 123) was conducted to compare the persuasive effects of inoculation messages (two-sided messages forewarning about misinformation related to COVID-19 vaccines), supportive messages (conventional health advocacy), and no message control. The results show that inoculation messages were superior to supportive messages at generating resistance to misinformation, as evidenced by more positive vaccine attitudes and stronger vaccine intention. Notably, while we expected the inoculation condition would produce more resistance than the control condition, there was little evidence in favor of this prediction. Attitudinal threat and counterarguing moderated the experimental effects; issue involvement and political trust were found to directly predict vaccine attitudes and intention. The findings suggest that future interventions focus on developing preventive mechanisms to counter misinformation and spreading inoculation over the issue is an effective strategy to generate resistance to misinformation. Interventions should be cautious about using health advocacy initiated by governments among populations with low political trust.

## Introduction

The World Health Organization (WHO) declared COVID-19 a global pandemic on March 12^th^, 2020 (Ghebreyesus, 2020). Following emergency use approvals, COVID-19 vaccines have been available in 92 countries (Bloomberg, 2021). These vaccines can slow the spread of novel coronavirus (Mukandavire et al., 2020; Voysey et al., 2021) and reduce hospital admissions and deaths (Cook and Roberts, 2021). While the widespread implementation of COVID-19 vaccines to reach herd immunity is the most promising way to end the pandemic (Randolph and Barreiro, 2020), different countries and regions have presented various forms of vaccine hesitancy (Dror et al., 2020; Lazarus et al., 2021), and some individuals refuse the vaccines outright (Murphy et al., 2021). These individuals express concerns over the efficacy and safety of the COVID-19 vaccines (Dror et al., 2020). The rapid development of vaccines and the use of new technologies (e.g., mRNA technology) have raised further uncertainties (Cimolai, 2020). One issue contributing to vaccine hesitancy and resistance is the spread of COVID-19 vaccine-related misinformation on the internet. Since the early stages of vaccine development, numerous examples of false information and conspiracy theories have been posted and shared on social networking sites. For example, there were claims that the vaccines were embedded with microchips, that the vaccines had been manufactured before the pandemic, and that the virus was human-made to increase vaccine sales (Islam et al., 2020; Agley and Xiao, 2021). These false claims could hinder the fight against the pandemic, as it has been found that exposure to COVID-19 vaccine misinformation reduces the intention to get vaccinated (Loomba et al., 2021).

Scientists have been urged to compete against, and overcome, this COVID-19 vaccine infodemic (Horton, 2020). The infodemic refers to an overabundance of information including false or misleading information in digital and physical environments during a disease outbreak (WHO, 2022). However, there are two major challenges in the fight against misinformation: first, as the internet reduces the cost of generating and disseminating information, false information and sensational stories are spread on a much quicker and larger scale (Wang et al., 2019). Health advocacies, normally initiated by healthcare professionals, scientists, communities, and media in a top-down manner, cannot compete with anti-vaccination messages as misinformation quickly influences the undecided groups *via* viral communication; whereas, verified information travels much slower (Vosoughi et al., 2018). The second issue is the fact that misinformation is encoded and memorized in relation to contextual factors—e.g., social networks and political orientation (Ecker et al., 2021)—when the individuals are first exposed to the misinformation. Post-hoc corrective efforts, such as removing false claims (Jin, 2020), usually fail to alter the mental representation and even increase familiarity with misinformation by simply repeating inaccurate statements (Lewandowsky et al., 2012; Swire et al., 2017).

One feasible way to fight misinformation is to use pre-debunking to build up resistance to the influence of misinformation in the first place (Van Bavel et al., 2020; van der Linden et al., 2020, 2021). Inoculation theory (McGuire, 1961) proposes that inoculating a listener with a two-sided, rebuttal message can forewarn individuals about the potential attack and generate resistance for attitude change when they are exposed to a subsequent attitude attack. The inoculation approach can effectively eliminate the influence of misinformation (Maertens et al., 2020). Recent empirical studies have also indicated that inoculation treatment could successfully protect against the negative effects caused by anti-vaccine messages and conspiracy theories (Wong, 2016; Jolley and Douglas, 2017).

The purpose of this study is to examine the ability of inoculation messaging to counter misinformation regarding the COVID-19 vaccines and to consider the impact of citizen trust in their government on this outcome. In Hong Kong, the context of this study, the implementation of COVID-19 vaccines was just over 10% at the initial stage (Government of Hong Kong Special Administrative Region, 2021; Wong et al., 2021), which is far lower than in many other countries (e.g., Israel, 57.7%; Chile, 29.2%; Bahrain, 28.9; the United States, 25.8%; Lazarus et al., 2021; Our World in Data, 2021). This low acceptance rate is potentially related to distrust of and dissatisfaction toward the Hong Kong government, which has increased in response to the Umbrella Movement in 2014 and the Anti-Extradition Bill Movement in 2019 (Chu and Yeo, 2020). On one hand, low political trust has impaired the credibility of health advocates and induced difficulties in the implementation of public health policies for COVID-19 (Min et al., 2020; Chau, 2021; Leung et al., 2021). On the other hand, this distrust has also motivated the spread of politicized discussions and misinformation about vaccination programs on social media platforms (Chou and Budenz, 2020; Reuters, 2021). To examine the effectiveness of inoculation, we conducted a three-phase experiment with Hong Kong university students to test if and in what circumstances inoculation messaging helps to guard against misinformation related to COVID-19 vaccines. This study’s findings contribute to the inoculation literature by providing evidence that inoculation messages can successfully inoculate against false information and preserve participants’ vaccine attitudes and intention. It also extends inoculation theory by revealing that belief threat and counterarguing moderate the effect of inoculation messaging. Additionally, these findings lend support to the claim that inoculation messages are a relatively effective strategy for countering false claims about the new COVID-19 vaccines.

## Countering the influence of misinformation using an inoculation approach

Inoculation theory begins with an assumption that people can learn to defend their existing attitudes against counter-attitudinal messages (Compton and Pfau, 2004; Parker et al., 2012; Richards et al., 2017). It posits that the attitudinal defense mechanism is similar to that which a body uses to protect itself from disease (O’Keefe and Nan, 2012). This theory, using the metaphor of medical inoculation, argues that attitudinal resistance to influence can be developed by introducing small doses of contrasting views in an attempt to minimize the persuasive effects of those perspectives at a later stage (McGuire, 1964). A two-sided message that includes a weakened version of a potential attack argument is considered to boost the production of rebuttal arguments, trigger a protective response after a period of incubation, and ultimately produce resistance. This may generate greater resistance to any strong persuasive attacks that are subsequently experienced.

Extensive studies have supported the effects of inoculation on persuasion. A meta-analysis of inoculation research (Banas and Rains, 2010) suggests that inoculation messages produce more resistance to subsequent attitudinal attacks than both supportive messages and no message controls. It is interesting that the inoculation does not have to refute the exact contrasting views that are encountered in order to produce attitude resistance. Refutational preemptions with the same or different inoculation are equally effective in conferring attitude resistance (Banas and Rains, 2010; Lee and Cameron, 2017). In addition, the use of inoculation is influential for groups with varying attitude stands—it protects the expected attitudes while shaping a neutral or antagonistic attitude towards the desired direction. Thus an inoculation approach can maximize persuasion effects and ensure that health practitioners do not have to rely on multiple strategies (Eisend, 2006; Wood, 2007; Banas and Rains, 2010).

While inoculation theory has been applied to a variety of issues in political and health communication (Compton and Pfau, 2005; Banas and Rains, 2010), it has recently been proposed as a promising approach for combating misinformation (Banas and Miller, 2013). As mentioned above, this psychological, persuasive mechanisms approach is superior to post-hoc corrects because it minimizes the impact of misinformation by warning the audience. It can effectively address variants of misinformation, as the audience has already developed “cognitive antibodies” for generation of refutational arguments (van der Linden et al., 2020). Last, inoculation theory is applicable to controversial issues like vaccination because it allows for the simultaneous influencing of groups with differing pre-attitudes. This study is the first to establish the superiority of the inoculation approach at countering any attitude change created by misinformation related to COVID-19 vaccines.

*H1*: Participants receiving the COVID-19 vaccine inoculation treatment designed to protect these attitudes will report (a) more positive attitudes towards the COVID-19 vaccine, (b) stronger intention to get the COVID-19 vaccine than those participants who receive a supportive or control message.

## Threat, refutational pre-emption and issue involvement in the inoculation process

Inoculation theory also posits that threat and refutational pre-emption are two necessary components for inducing resistance to subsequent counterattitudinal persuasion (Compton and Pfau, 2005). The threat element, defined as the recognition or perception that an attitudinal challenge may be impending, will likely “trigger the receiver’s motivation to bolster attitudes and gives inoculation its distinctive power” (Pfau, 1995, p. 101). Perceived attitudinal threat is theorized to stimulate the defense mechanism to the contrasting persuasion, and greater threats are associated with more resistance (Compton and Pfau, 2005). Although previous meta-analysis (Banas and Rains, 2010) did not statistically establish threat as a moderator, presumably due to lack of statistical power, several studies have offered qualitative evidence that the inoculation effects were stronger when the audience perceived more attitudinal threat (Pfau et al., 2005; Silvia, 2006).

*H2*: Attitudinal threat is a moderator in the relationships between message condition and (a) vaccine attitudes, and between message condition and (b) intention to get the COVID-19 vaccine.

The second component in inoculation theory is refutational preemption, which is the activation of one’s counterargument for future defense (Pfau et al., 1997). Refutational preemption can be further classified as active refutation and passive refutation. The active refutational approach asks the audience to craft arguments to defend their attitudes while the passive refutational approach provides the audiences with specific content that they can use to argue against subsequent attacks (Pfau et al., 1997). Common practices combine these by providing the rebuttal materials and by inviting the audience to practice counterarguments. With practice, audiences find it easier to dispute the arguments presented in attitudinal attacks, and counterarguments further strengthen attitude confidence and resistance to attacks (Clear et al., 2021).

*H3*: Counterarguing is a moderator in the relationships between message condition and (a) vaccine attitudes, and between message condition and (b) intention to get the COVID-19 vaccine.

The effectiveness of inoculation strategies also varies according to issue involvement, which can be defined as the personal importance one gives to an issue and its consequences (Pfau et al., 2009a). It has been argued that high issue involvement is associated with more elaboration when processing an inoculation message, leading to more resistance to attitudinal attacks (Pfau et al., 1997). Although previous meta-analysis did not find issue involvement to be a significant moderator (Banas and Rains, 2010), some studies have indicated that inoculation creates more attitudinal resistance among highly involved individuals (Cornelis et al., 2014).

*H4*: Involvement is a moderator in the relationship between message condition and (a) vaccine attitudes, and between message condition and (b) intention to get the COVID-19 vaccine.

## Materials and methods

### Design

The hypotheses were examined by a three-phase, 1 × 3 between-subjects experiment. In the first phase, the participants were pre-tested for their demographic information, issue involvement, and pre-attitudes toward COVID-19 vaccines. In the second phase, they were randomly assigned to read an inoculation message, supportive message, or no message (control), and then assessed for vaccine attitudes and intention and checked for manipulation. In the third phase, all the participants are exposed to an attack message that used a set of conspiracies to argue against COVID-19 vaccines and assessed again for vaccine attitudes and intention. As the experiment was conducted in 2020 when the mature vaccines against COVID-19 had not been yet available in Hong Kong, all the participants in the present study were not vaccinated.

### Participants

A total of 196 undergraduate students from the two universities in Hong Kong were recruited to participate in the study. They were compensated with either 1.5 extra course credits or a $100 supermarket cash coupon. The retention rate was 62.76% over the three experimental phases, resulting in a final sample of 123 participants (*n* = 123).

The majority of the participants were female students (*n* = 77, 62.6%) and their age ranged from 17 to 26 years (*M* = 19.94, *SD* = 1.59). Most of them were local residents of Hong Kong (*n* = 119, 96.7%). Most had been infected with influenza (*n* = 97, 78.9%) and had taken influenza vaccine before (*n* = 103, 83.7%). The human ethical review committee at the authors’ affiliated University approved the data collection.

### Procedure

A secure online survey platform (Qualtrics) collected the data for this three-phase experiment. In Phase 1, participants’ demographic information was collected (age, sex, education, location of residence, political orientation, political trust, and vaccination experience). Pre-attitudes toward vaccines were also evaluated. At the end of Phase 1, participants were randomly assigned to one of the three conditions (i.e., control group, supportive message group, and inoculation message group).

There was a one-week delay between Phases 1 and 2. Participants were then emailed a link that led them to the assigned condition in Phase 2. In the supportive condition, participants were exposed to a news article that advocated COVID-19 vaccines; in the inoculation condition, participants were exposed to a news article that lightly refuted a COVID-19 vaccine conspiracy; in the control condition, participants received no message treatment. At the end of Phase 2, all participants were assessed for their attitudes toward COVID-19 vaccines and their intention to get a vaccine. They were also asked to rate their perceptions of attitudinal threat, and to counter-argue against the opinions that are different from their standing points in an open-ended question. In the two message conditions (inoculation and supportive), the participants were assessed for their negative emotions after reading the message.

In Phase 3, all the participants were exposed to an attack message that extensively argued against COVID-19 vaccines using several conspiracy theories 1 week after they were exposed to the treatment in Phase 2. They then completed post-test measures and were debriefed at the end of the experiment. In the debriefing, participants were informed that the news reports described rare incidents of severe adverse effects, and they were reassured that COVID vaccines have been scientifically tested and that their safety is continually monitored.

### Stimuli messages

#### Inoculation and supportive messages

During Phase 2, we manipulated two treatment messages, an inoculation message and a supportive message. Following previous research (Pfau et al., 2005; Ivanov et al., 2009a; Miller et al., 2013), the inoculation message started with a paragraph aimed at generating explicit threat by cautioning the participants of an impending attack on their attitudes about the COVID-19 vaccines:

“Many reports and stories by the media and various interest groups are aimed at attacking your attitude and feelings on this issue, and there is a real possibility that you will come into contact with these arguments in the near future, some of which are so persuasive that they may cause you to question your attitude and feelings toward getting the COVID-19 vaccine.”

The threat message is crafted based on those used in past inoculation studies (e.g., Ivanov et al., 2009a; Pfau et al., 2009b). The message was followed with a refutational pre-emption component, which briefly listed several conspiracy theories that tarnish the safety and efficacy of the COVID-19 vaccines and then, respectively, dispelled these conspiracy theories with evidence. The message also provided reassurance of the safety and efficacy of the COVID-19 vaccines: very few adverse effects to the vaccine have been reported from the millions of doses given, the vaccine is approved and endorsed by a variety of international and domestic medical authorities, and it has been shown to prevent infections in scientific studies.

The supportive message took the format of conventional vaccine advocacy and was modified based on the webpage of Centre for Health Protection in Hong Kong on influenza vaccines. The message listed several reasons to cultivate positive attitudes toward the COVID-19 vaccines and suggest the audience consider getting vaccinated when possible. Both treatment messages were presented in the format of a news article published by a politically-neutral newspaper (MingPao) to avoid any possible influence from the media’s political standing (Zhu et al., 2017). The two messages were also carefully crafted to ensure similar lengths and tones.

#### Attack messages

The attack message used in Phase 3 was presented as a news article published by another politically-neutral newspaper (Sing Pao Daily). Modified based on real anti-vaccine content, the message featured the big pharma conspiracy theory that claims the medical community and big pharmaceutical companies advocate compulsory vaccination for profits rather than for the public good. To enhance the attack, the message also quoted several myths or rumors from real news stories, including the fraudulent association between the MMR (measles, mumps and rubella) vaccine and autism, unconfirmed safety and efficacy, and the Bill Gates microchip conspiracy.

### Measures

*Vaccine attitudes* were measured using six bipolar items adapted from Burgoon et al. (1978). On a 7-point scale, the participants rated the following statement: “your overall attitudes toward getting the COVID-19 vaccine are negative/positive, wrong/right, foolish/wise, good/bad, unfavorable/favorable, and unacceptable/acceptable.” The scores were averaged to form an index for vaccine attitudes, with higher scores indicating more positive attitudes toward the COVID-19 vaccine (for Phase 1, Cronbach’s α =0.95, 95% CI = [0.94, 0.96]; for Phase 2, Cronbach’s α =0.96, 95% CI =; for Phase 3, Cronbach’s α =0.97, 95% CI =).

*Vaccination intention* was measured by a 7-point scale adapted from Wong (2014). The respondents were asked to respond from 1 (not at all) to 7 (very likely) to three items: (1) “I’m seriously considering getting the COVID-19 vaccine in the next 6 months,” (2) “It’s likely that I intend to get the COVID-19 vaccine in the next 6 months,” and (3) “I will exert much effort to get the COVID-19 vaccine in the next 6 months.” The scores were averaged to form an index score; higher scores indicated stronger behavioral intention (for Phase 2, Cronbach’s α =0.96, 95% CI =; for Phase 3, Cronbach’s α =0.96, 95% CI =).

*Issue involvement* was measured by six items adapted from the Personal Involvement Inventory (Zaichkowsky, 1985). The items asked the participants to rate, on a 7-point scale, the issue of the COVID-19 vaccine as unimportant/important, of no concern/of much concern, superfluous/vital, insignificant/significant, trivial/fundamental, or irrelevant/relevant (Cronbach’s α = 0.95; 95% CI = [0.94, 0.96]).

*Political trust* was measured in Phase 1 by asking respondents to rate their trust in four authorities in Hong Kong on a 7-point scale (1 = totally distrust, 7 = totally trust; Marien and Hooghe, 2011). These authority figures were the government, justice systems, the police, and public health institutions. The scores were averaged, with higher scores indicating higher levels of trust (Cronbach’s α = 0.88; 95% CI = [0.85, 0.91]).

*Perceived threat* was evaluated in Phase 2 by a 7-point scale, with six items composing bipolar adjectives: nonthreatening/threatening, not harmful/harmful, not dangerous/dangerous, not risky/risky, calm/anxious, and not scary/scary. This scale had been successfully applied in previous inoculation research (e.g., Pfau et al., 1992, 2005; Ivanov et al., 2009b). The scores were averaged, with higher scores indicating higher levels of perceived threat (Cronbach’s α =0.94, 95% CI = [0.92, 0.96]).

*Negative emotions* were measured after the message treatment in Phase 2 and after the attack messages in Phase 3 using four questions adapted from Nabi (2003). On a scale of 1 to 7, the respondents were asked to rate how strongly they felt—angry, annoyed, irritated, and aggravated—while watching the message they had just seen. The six emotions formed a single-factor index representing negative emotional arousal (for Phase 2, Cronbach’s α =0.97, 95% CI = [0.96, 0.98]; for Phase 3, Cronbach’s α =0.94, 95% CI = [0.92, 0.95]).

*Counterarguing Output* was measured in Phase 2 by utilizing a thought-listing procedure (Brock, 1967). This procedure, widely used in previous inoculation studies (e.g., Pfau et al., 1997), is a typical procedure of cognitive response evaluation. The open-ended cognitive assessment techniques help to illuminate the cognitive structures and processes underlying various clinical problems (Cacioppo et al., 1997). The participants were presented with the question, “Suppose you encounter information that is different from your attitude towards vaccines and makes you feel that your attitude is threatened. How would you refute it? (Please write down the basis of your refutation in detail).” Because most participants generated one or no thought in their counterargument output, we instead adjusted the counterarguing variable and categorized it as anti-vaccine, neutral, or pro-vaccine. Two of the authors independently coded each participant-generated argument into three categories (−1 = anti-vaccine, 0 = neural, 1 = pro-vaccine; Krippendorff’s Alpha = 0.87) and counted the total number of arguments generated by each participant. Then the third author discussed with the two authors to reach an agreement for each argument category. The anti-vaccine category included arguments that expressed concerns and reservations over the vaccine (e.g., “I would argue that the safety of the vaccine is uncertain”). The neural category included arguments that did not indicate an obvious preference (e.g., “I do not think I will refute it because it depends on personal choice, and I respect that)” or no thoughts. The pro-vaccine category included arguments that favored COVID-19 vaccination (e.g., “It is believed that vaccines are relatively the best treatment of COVID-19, they have a higher chance to protect people from being infected and cure the illness”).

#### Analyses

We used SPSS 25.0 to perform the statistical analyses in this study. We first checked for the assumptions of uncorrelated errors, homoscedasticity, collinearity, and normality in our main measures. All the assumptions were met (see Supplementary material). Then, Pearson correlation was conducted to show the zero-order correlation matrix of the variables. Next, a paired t-test was conducted to test the differences in vaccine attitude for pre-attack and post-attack. In addition, a multivariate analysis of covariance (MANCOVA) was performed on the dependent variables (vaccine attitudes and vaccine intention) to examine the effects of the experimental condition. Finally, moderation analyses were conducted using the PROCESS models in SPSS.

## Results

### Manipulation checks

Table 1 presents the means, standard deviations, and correlation matrix of the main variables in the study. We validated our message manipulation by assessing group differences regarding perceived threat, negative emotion, and counterargument output in Phase 2. It was found that the perceived threat in the inoculation group (*M* = 4.50, *SE* = 0.19) was higher than in the supportive group (*M* = 3.80, *SE* = 0.19) and control group (*M* = 3.70, *SE* = 0.19), *F*(2,133) = 5.22, *p* = 0.007. The participants in the inoculation group experienced more negative emotions (*M* = 3.56, *SE* = 0.24) than those in the supportive group (*M* = 2.18, *SE* = 0.24), *t*(88) = −4.08, *p* = 0.001. However, there was low evidence against the null hypothesis of no difference in counterargument output, *F*(2,133) = 0.56, *p* = 0.57. In other words, we did not find strong evidence in favor of the expected differences in the number of arguments that they generated. While McGuire (1961) suggests that counterarguing is one of the resistance-induction mechanisms, studies have shown that inoculation can generate resistance without the definitive evidence of counterarguments (Compton and Pfau, 2005; Compton and Ivanov, 2012; Ivanov, 2017). Thus, provided that the manipulation of perceived threat and negative emotion was successful, we proceeded to test the hypotheses.

We also validated whether the attack was successful by comparing the persuasive outcomes on the control group before and after the attack manipulation. A paired *t*-test revealed low evidence of the null hypothesis of no differences in vaccine attitude [for pre-attack, *M* = 5.40, *SE* = 0.21; for post-attack, *M* = 4.98, *SE* = 0.15; *t*(42) = 2.39, *p* = 0.033] and vaccine intention [for pre-attack, *M* = 4.35, *SE* = 0.19; for post-attack, *M* = 3.74, *SE* = 0.20; *t*(42) = 3.37, *p* = 0.002]. Thus, the attack message was sufficiently persuasive to warrant its use in this study.

**Table 1:** Zero-order correlation matrix of the variables.

	*M(SD)*	1	2	3	4	5	6	7	8	9	10	11	12	13	14
1. Gender	/	1													
2. Age	19.94(1.59)	0.13	1												
3. Residence location	/	0.00	−0.03	1											
4. Influenza history	/	0.08	0.08	0.07	1										
5. IV history	/	−0.08	0.13	−0.10	0.03	1									
6. Political trust	2.9(1.21)	0.09	−0.08	0.21^**^	0.08	0.02	1								
7. Involvement	5.19(1.22)	0.00	−0.05	0.16	−0.08	−0.15	0.20^*^	1							
8. Perceived threat	3.99(1.31)	0.15	−0.15	0.03	0.02	−0.06	−0.07	−0.11	1						
9. Counterarguing	0.87(0.47)	0.10	0.10	0.05	0.04	−0.10	0.20^*^	0.38^***^	−0.19^*^	1					
10. Phase 1 Attitude	5.18(1.28)	−0.12	−0.10	0.20^**^	−0.06	−0.14	0.34^**^	0.58^**^	−0.16	0.10	1				
11. Phase 2 Attitude	5.05(1.13)	0.03	−0.11	0.16	−0.13	−0.14	0.26^**^	0.77^**^	−0.12	0.20^*^	0.57^**^	1			
12. Phase 3 Attitude	4.80(1.14)	−0.00	0.06	0.25^**^	−0.04	0.13	0.28^**^	0.72^***^	0.14	0.20^*^	0.58^**^	0.77^**^	1		
13. Phase 2 Intention	4.06(1.57)	0.07	−0.10	0.15	−0.05	−0.19^*^	0.33^**^	0.66^**^	−0.03	0.11	0.44^**^	0.70^**^	0.63^**^	1	
14. Phase 3 Intention	3.76(1.54)	0.10	−0.09	0.24^**^	0.07	−0.22	0.38^***^	0.59^***^	−0.09	0.08	0.44^**^	0.66^**^	0.68^**^	0.75^**^	1

For residence location, 1 = Hong Kong, 2 = Chinese mainland, 3 = others. IV history = Influenza vaccination history.

* *p* < 0.05;

** *p* < 0.01;

*** *p* < 0.001.

### Hypothesis testing

H1 predicted the participants receiving the inoculation message would report more positive attitudes and stronger intention to get the COVID-19 vaccine. To test this, a multivariate analysis of covariance (MANCOVA) was performed on the dependent variables (vaccine attitudes and vaccine intention) to examine the effects of the experimental condition (see Table 2). The Bonferroni corrections were used for multiple comparisons of means. There was strong evidence against the null hypothesis for vaccine attitudes [*F*(2, 113) = 4.79, *p* = 0.010, *η^2^* = 0.08] and vaccine intention [*F*(2, 113) = 4.30, *p* = 0.016, *η^2^* = 0.07]. Pairwise comparisons suggested that the inoculation group reported more positive vaccine attitudes (*p* = 0.045; Cohen’s d = 0.43) and stronger vaccination intention (*p* = 0.005; Cohen’s d = 0.62) than the supportive group. The supportive group reported less positive vaccine attitudes (*p* = 0.003; Cohen’s d = 0.73) and weaker intention (*p* = 0.035; Cohen’s d = 0.54) than the control group. However, the results provided little evidence against the difference between the control and inoculation groups regarding vaccine attitudes (*p* = 0.45) or vaccine intention (*p* = 0.24). Thus, H1 was partially supported. The effect sizes observed ranged from small effects to intermedia effects (Cohen, 1988), consistent with those documented in the previous meta-analysis of inoculation effects (Banas and Rains, 2010).

**Table 2:** Mean comparisons for dependent measures as a function of experimental conditions.

	Supportive	Inoculation	Control
Dependent measure	(*n* = 41)	(*n* = 39)	(*n* = 43)
Attitudinal threat	3.80 (0.19)^a^	4.50 (0.19)^b^	3.70 (0.19)^a^
Counterarguing	0.46 (0.11)^a^	0.30 (0.11)^a^	0.43 (0.11)^a^
Phase 3 Vaccine attitude	4.06 (0.19)^a^	4.58 (0.16)^b^	4.85(0.17)^b^
Phase 3 Vaccine intention	2.86 (0.27)^a^	3.86 (0.23)^b^	3.62 (0.23)^b^

All of the dependent variables except for counterarguing were measured using 7-point scales. Pretest measures for attitudes and intention regarding the COVID-19 vaccine were controlled for as covariates. Different subscripts (e.g., ^a,b^) within rows are significantly different at *p* < 0.05 based on Bonferroni-adjusted pairwise comparisons.

**Figure 1:**
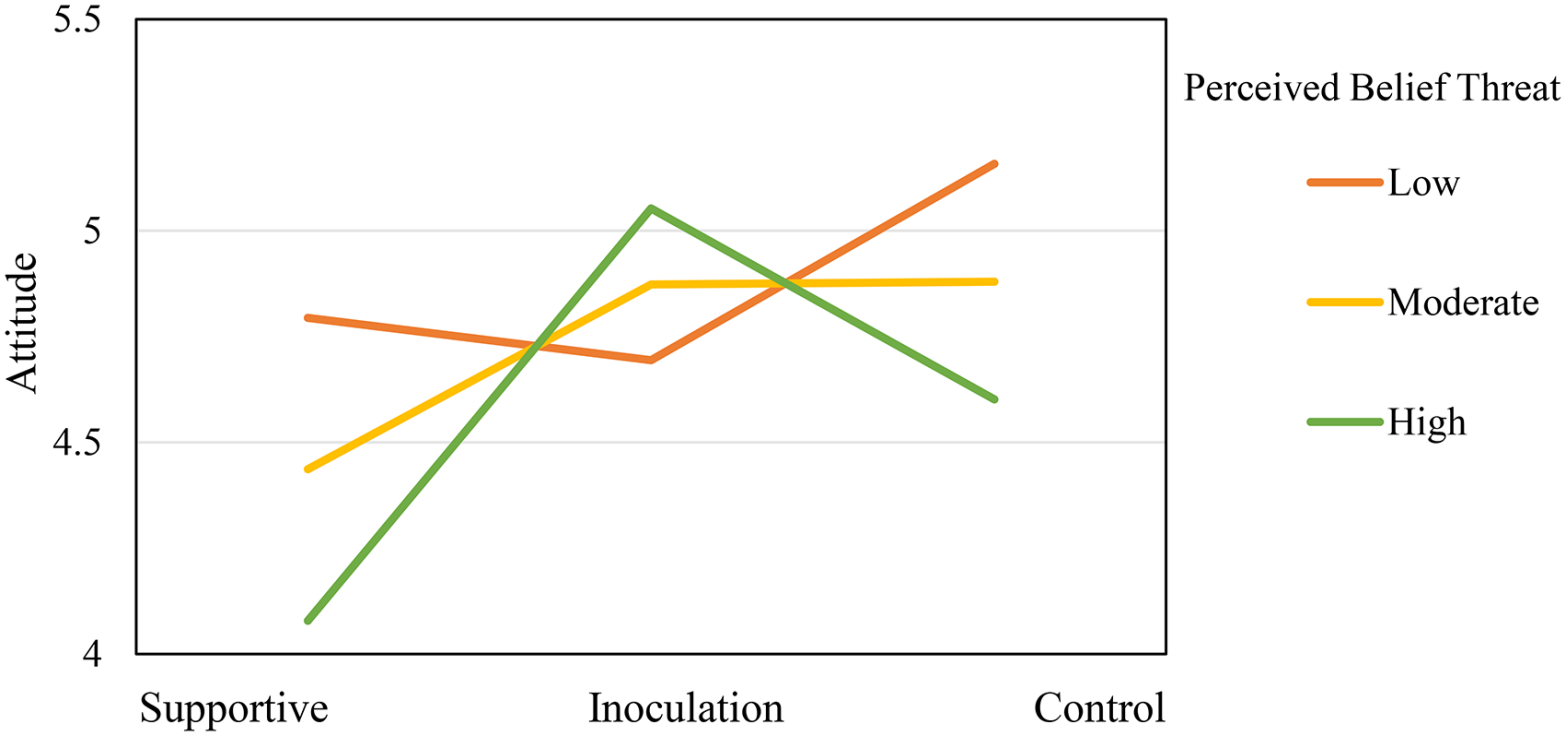
The moderating effect of perceived belief threat among the three groups.

It was hypothesized that attitudinal threat (H2), counterarguing (H3), and issue involvement (H4) would moderate the experimental effects. We, respectively, tested each moderator in the PROCESS models in SPSS (see Table 3). First, it was found that attitudinal threat moderated the relationship between condition and attitudes. The test of interaction effect (condition x threat) provided strong evidence against the null hypothesis of equal experimental effects at different threat levels: B = 0.36, *SE* = 0.17, *t* = 2.09, *p* = 0.039. As indicated by Figure 1, the difference in vaccine attitudes between the inoculation group and the supportive group was largest when the participants reported a high level of attitudinal threat. However, attitudinal threat did not moderate the experimental effects on vaccine intention. Thus, H2 was partially supported.

**Table 3:** Moderation analysis.

	Vaccine Attitude			Vaccine Intention		
	B	SE	*t* value	B	SE	*t* value
Inoculation (X1)	−1.21	0.73	−1.67	−0.42	1.03	−0.41
Control (X2)	0.20	0.75	0.27	1.02	1.05	0.96
Threat (W)	−0.28^*^	0.13	−2.07	−0.27	0.19	−1.46
X1 x W	0.41^*^	0.17	2.45	0.39	0.24	1.64
X2 x W	0.06	0.19	0.32	−0.12	0.27	−0.45
Inoculation (X1)	0.75	0.59	1.27	0.37	0.82	0.46
Control (X2)	1.64^**^	0.60	2.72	1.46	0.83	1.75
Neural arguing (W1)	1.55^**^	0.55	2.83	1.27	0.77	1.65
Pro-vaccine arguing (W2)	2.11^***^	0.52	4.04	1.54^*^	0.74	2.09
X1 x W1	−0.77	0.70	−1.11	0.60	0.98	0.61
X1 x W2	0.10	0.66	0.14	1.27	0.93	1.37
X2 x W1	−1.21	0.72	−1.68	−1.40	0.99	−1.41
X2 x W2	−1.40^*^	0.67	−2.10	−0.72	0.93	−0.77
Inoculation (X1)	0.37	0.71	0.52	−0.40	1.12	−0.36
Control (X2)	−0.56	0.77	−0.72	1.43	1.24	1.16
Involvement (W)	0.56^***^	0.91	6.24	0.69^***^	0.15	4.67
X1 x W	−0.05	0.13	−0.34	−0.24	0.23	−1.05
X2 x W	0.12	0.14	0.85	−0.42	0.26	−1.67

For experimental conditions (X), supportive group was the reference group; X1 denotes the comparison between inoculation and supportive groups; X2 denotes the comparison between control and supportive groups. For counterarguing category, anti-vaccine arguing was the reference group; W1 denotes the comparison between neutral and anti-vaccine arguing; W2 denotes the comparison between pro-vaccine and anti-vaccine arguing.

* *p* < 0.05;

** *p* < 0.01;

*** *p* < 0.001.

**Figure 2:**
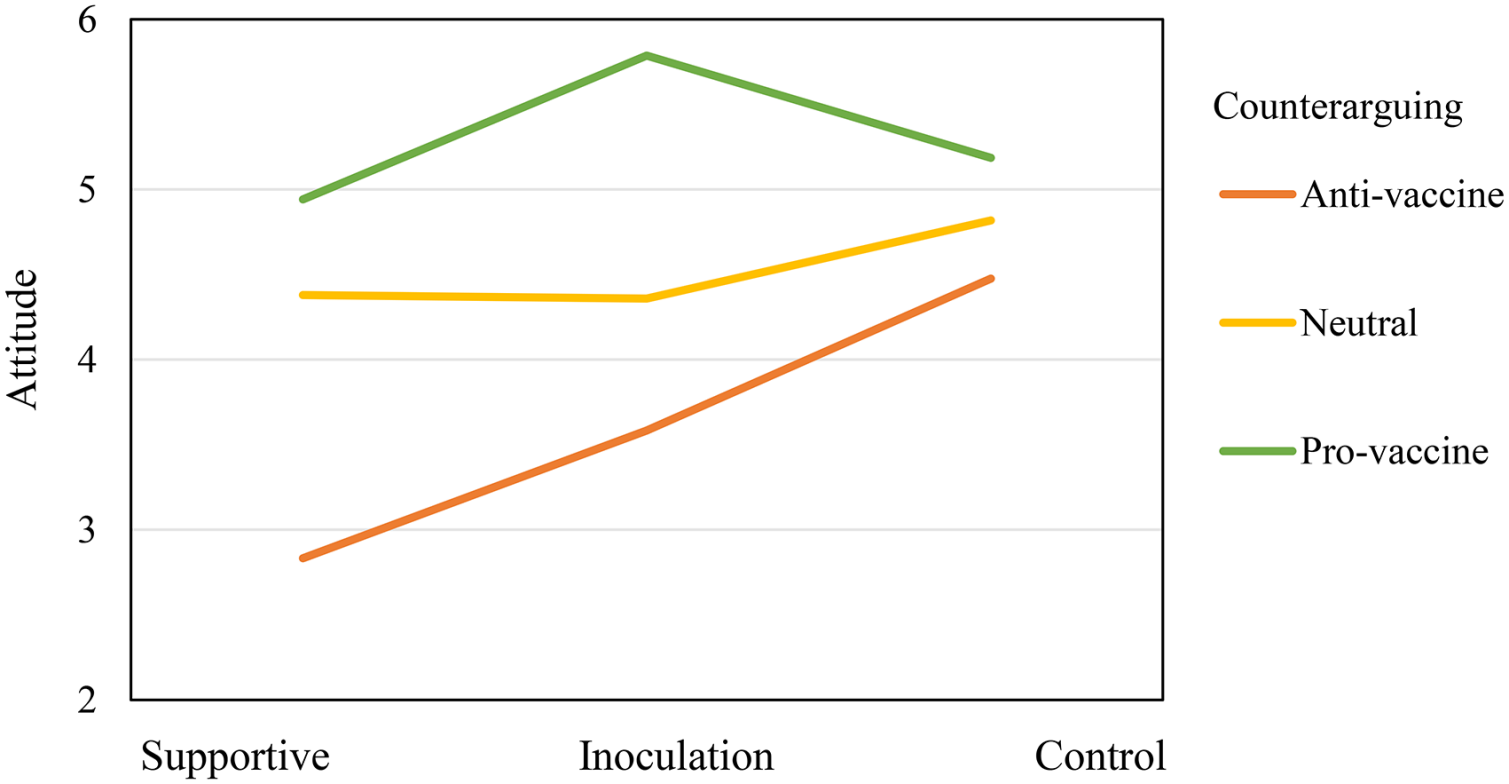
The moderating effect of counterarguing among the three groups.

Counterarguing category moderated the inoculation effect on attitudes because there was strong evidence against the null hypothesis of equal experimental effects at different counterarguing categories: B = −1.40, *SE* = 0.67, *t* = −2.10, *p* = 0.038. As indicated by Figure 2, while the control group demonstrated a more positive vaccine attitude than did the supportive group after receiving the attack message in Phase 3 (B = 1.64, *SE* = 0.60, *t* = 2.72, *p* = 0.008), there was little support for this difference among the participants who counterargued in favor of vaccines. Counterarguing category did not moderate the experimental effects on vaccine intention. Thus, H3 was partially supported. At the same time, there was a main effect of counterarguing on vaccine attitudes and intention. Making a pro-vaccine argument in the counterargument increased the positivity of the participants’ vaccine attitudes (B = 2.11, SE = 0.52, *t* = 4.03, *p* = 0.0001) and strengthened their intention (B = 1.54, SE = 0.73, *t* = 2.78, *p* = 0.039; See Table 3).

Issue involvement did not moderate the experimental effects on attitudes and intention (see Table 3). Thus, H4 was not supported. However, issue involvement was positively associated with vaccine attitude (B = 0.56, SE = 0.09, *t* = 6.24, *p* = 0.00001) and intention (B = 0.69, SE = 0.15, *t* = 4.67, *p* = 0.0001). Political trust was positively associated with vaccine intention (B = 0.52, SE = 0.20, *t* = 2.60, *p* = 0.011).

## Discussion

This study tested inoculation theory within the context of COVID-19 vaccination. According to the results of this three-phase experiment, participants who received inoculation messages reported higher vaccine attitudes and vaccine intention than did those in the supportive condition. The study, however, found little evidence for expected differences between the inoculation and control conditions on the outcome variables. Consistent with our predictions, both attitudinal threat and counterarguing moderated the relationships between the experimental conditions and the outcome variables. Notably, although it was hypothesized that issue involvement would act as a moderator, the results did not provide any support for this hypothesis.

To the best of our understanding, the present research is one of the early applications of inoculation theory on vaccine issues. While prior empirical studies have applied inoculation theory to various health-related issues, including alcohol consumption and legalization of marijuana, there were only a few empirical studies that applied inoculation theory to the vaccine issue (Compton et al., 2016; Wong, 2016; Clear et al., 2021). In this experiment, we examined the effect of inoculation treatment on participants who subsequently encountered COVID-19 vaccine conspiracy theories. In line with the previous studies in the vaccine context, our findings indicate that providing inoculation treatment prior to the exposure of vaccine-related misinformation could protect individuals’ vaccine attitudes and intention. This study provides additional evidence to support the theory that presentation of a pre-debunking message can effectively shield individuals from the negative influence of vaccine conspiracy theories and misinformation and thus should be applied to counter the current flood of COVID-19 vaccine misinformation (van der Linden et al., 2020, 2021).

These results indicate that perceived threat and counterargument moderated the relationship between experimental conditions and resistance to attitude change regarding COVID-19 vaccines, which supports the tenets of inoculation theory. In addition to perceived threat and counterargument, scholars have proposed that issue involvement would also intervene in the effect of inoculation on resistance. However, consistent with the meta-analytic review of Banas and Rains (2010), this experiment found little evidence for the moderating effect of issue involvement. Although it is possible that the effect of inoculation messaging on resistance regarding a specific issue is not dependent on involvement, another explanation is that we examined the involvement of a particular topic, i.e., the COVID-19 vaccine, instead of manipulating involvement in the messages as proposed by Banas and Rains (2010). Also, we suspect that the majority of our participants are highly involved in the issue because to enter university campuses, students were required to be vaccinated or undergo self-paid antigen tests weekly. This characteristic may have led to our inability to detect any potential moderating effect of issue involvement.

Additionally, health promotion should be cautious about the use of supportive messages to advocate vaccination. While supportive messaging has been utilized to promote the adoption of vaccines, our study revealed that it could potentially backfire. Informed by the previous inoculation research, we originally hypothesized that there would be higher resistance in inoculation condition than in either the control or the supportive conditions, and that the control and supportive conditions would produce equivalent outcomes. Nonetheless, the current findings indicate that, after receiving the attack message, individuals in the supportive condition reported lower vaccine attitudes and weaker intention than did those in either the inoculation or the control conditions. We believe that both issue involvement and political trust may play a role in this finding because issue involvement was found to be positively associated with vaccine attitude and intention, while political trust was found to be positively associated with vaccine intention. The issue of COVID-19 vaccination has been a widespread concern amongst many local university students and, given the intensive exposure to COVID-19 vaccine information by the media, they might not have paid much attention to the supportive message. On the other hand, students with low political trust might not support or believe in the supportive message, as it parallels the public opinions of the authorities. Instead, due to their limited trust in the authorities, they might be more likely to believe the conspiracy theories, which would result in a potential backfire effect from the supportive message. In sum, as participants in the supportive condition presented even lower vaccine attitudes and weaker intention than did those in the control condition, there is some evidence that the implementation of supportive vaccine messaging could ultimately make the receivers more vulnerable to conspiracy theories and misinformation.

### Practical implications

This study has two major practical implications. First, our findings support the hypothesis that a preventive approach is superior to a cure approach when combating misinformation. Especially in regard to health misinformation, the most common approach is to correct and debunk misinformation through use of expert opinion and scientific evidence. However, correcting real-world misinformation is exceptionally challenging, and it has been suggested by a recent meta-analysis that the effect of correction on belief is weak (*r* = 0.14; Walter and Murphy, 2018). Further, it can be even more difficult to debunk misinformation once the audience begins to craft support for specific claims of that misinformation (Chan et al., 2017). The effectiveness of a post-hoc approach is therefore likely to be inadequate. Instead, the current findings reveal that individuals who received COVID-19 vaccine inoculation treatment were able to preserve their positive vaccine attitudes and strong vaccine intention after being exposed to the attack message, indicating that inoculation messaging could potentially protect individuals from future misinformation. Therefore, a switch from post-hoc correction to inoculation might help to limit the negative impact of false information.

Second, the findings of this study could inform the creation of a low-cost, theory-based social media campaign to increase resistance to vaccine-related misinformation and improve the uptake rates of vaccines among high-risk groups. Communication scholars have proposed that the inoculation approach can be integrated with word-of-mouth communication and interpersonal processes, such as post-inoculation talk, and that social sharing could help maximize the persuasive effects produced by inoculation strategies (Compton and Pfau, 2005; Compton and Ivanov, 2012). Besides supporting the effectiveness of inoculation treatment that has been found in the prior research, our study further identified attitudinal threat and counterarguing as moderators that influence the effect of inoculation on resistance. This information could be beneficial to health experts as they design pertinent social media campaigns that aim to protect individuals from future vaccine-related misinformation and increase vaccination rates.

### Limitations and future directions

This study has several limitations that indicate potential avenues for future research. One of the limitations is related to the counterarguing measures. Consistent with prior studies of inoculation, we employed a thought-listing procedure to measure participants’ levels of counterarguing by examining their refutation of vaccine-related opposing views. Although the participants were encouraged to write down their refutations in detail, many of the participants, unfortunately, provided relatively short statements or even no response to the question. This created challenges in assessing and categorizing respondents’ level of counterarguing. Future studies should explore other appropriate measures or consider using the existing counterarguing scales (e.g., Silvia, 2006; Nabi et al., 2007).

The second limitation of this study concerns the stimuli that were utilized. Conspiracy theories were used in both the inoculation message and the attack message, both of which were presented to the participants through fictional news articles. The sole use of conspiracy theories and a single format to present the message may have limited the generalizability of our findings. Different forms of message design, as well as different types of false information, should be tested in future studies.

Another limitation worth mentioning concerns the sample of the study. We recruited Hong Kong university students as participants, and most were local residents. To the local university students, COVID-19 vaccination is a high-involvement issue; most expressed some level of concern about the issue. In addition, it was found that the majority of the participants reported low political trust. Given the high level of issue involvement and low political trust reported by the participants, this study’s findings might not be applicable to the general population.

Lastly, the current study measured only vaccine intention as opposed to actual vaccination behavior. Further investigations are needed to evaluate the effectiveness of inoculation strategy in promoting vaccination behaviors.

## Data availability statement

The raw data supporting the conclusions of this article will be made available by the authors, without undue reservation.

## Ethics statement

The studies involving human participants were reviewed and approved by City University of Hong Kong. The patients/participants provided their written informed consent to participate in this study.

## Author contributions

All persons who meet authorship criteria are listed as authors, and all authors certify that they have participated sufficiently in the work to take public responsibility for the content, including participation in the concept, design, analysis, writing, or revision of the manuscript. Furthermore, each author certifies that this material or similar material has not been and will not be submitted to or published in any other publication before its appearance.

## Funding

All sources of funding received for the research have been submitted. The two research grants from the City Univesity of Hong Kong (Project Number: 7020009/9618020) were awarded to LJ (PI) and the Public Policy Research Fund 2020-21 (Project Number: 2020.A1.093.20A) was awarded to SC (PI) and LJ (Co-PI).

## Conflict of interest

The authors declare that the research was conducted in the absence of any commercial or financial relationships that could be construed as a potential conflict of interest.

## Publisher’s note

All claims expressed in this article are solely those of the authors and do not necessarily represent those of their affiliated organizations, or those of the publisher, the editors and the reviewers. Any product that may be evaluated in this article, or claim that may be made by its manufacturer, is not guaranteed or endorsed by the publisher.

## References

[ref1] Agley J. Xiao Y. (2021). Misinformation about COVID-19: evidence for differential latent profiles and a strong association with trust in science. BMC Public Health 21:89. doi: 10.1186/s12889-020-10103-x, PMID: 33413219 PMC7789893

[ref2] Banas J. A. Miller G. (2013). Inducing resistance to conspiracy theory propaganda: testing inoculation and metainoculation strategies. Hum. Commun. Res. 39, 184–207. doi: 10.1111/hcre.12000

[ref3] Banas J. A. Rains S. A. (2010). A meta-analysis of research on inoculation theory. Commun. Monogr. 77, 281–311. doi: 10.1080/03637751003758193

[ref5] Bloomberg (2021). More than 205 million shots given: COVID-19 tracker. Available at: https://www.bloomberg.com/graphics/covid-vaccine-tracker-global-distribution/ (Accessed June 6, 2022).

[ref6] Brock T. C. (1967). Communication discrepancy and intent to persuade as determinants of counterargument production. J. Exp. Soc. Psychol. 3, 296–309. doi: 10.1016/0022-1031(67)90031-5

[ref7] Burgoon M. Cohen M. Miller M. D. Montgomery C. L. (1978). An empirical test of a model of resistance to persuasion. Hum. Commun. Res. 5, 27–39. doi: 10.1111/j.1468-2958.1978.tb00620.x

[ref8] Cacioppo J. T. von Hippel W. Ernst J. M. (1997). Mapping cognitive structures and processes through verbal content: the thought-listing technique. J. Consult. Clin. Psychol. 65, 928–940. US: American Psychological Association. doi: 10.1037/0022-006X.65.6.928, PMID: 9420354

[ref9] Chan M.-P. S. Jones C. R. Hall Jamieson K. Albarracín D. (2017). Debunking: a meta-analysis of the psychological efficacy of messages countering misinformation. Psychol. Sci. 28, 1531–1546. doi: 10.1177/0956797617714579, PMID: 28895452 PMC5673564

[ref10] Chau C. (2021). Covid-19: Some Hongkongers shun gov’t tracking app over privacy concerns as new rules rolled out at eateries. Hong Kong Free Press. Available at: https://hongkongfp.com/2021/02/19/covid-19-some-hongkongers-shun-govt-tracking-app-over-privacy-concerns-as-new-rules-rolled-out-at-eateries/ (Accessed June 6, 2022).

[ref11] Chou W.-Y. S. Budenz A. (2020). Considering emotion in COVID-19 vaccine communication: addressing vaccine hesitancy and fostering vaccine confidence. Health Commun. 35, 1718–1722. doi: 10.1080/10410236.2020.1838096, PMID: 33124475

[ref12] Chu T. H. Yeo T. E. D. (2020). Rethinking mediated political engagement: social media ambivalence and disconnective practices of politically active youths in Hong Kong. Chin. J. Commun. 13, 148–164. doi: 10.1080/17544750.2019.1634606

[ref13] Cimolai N. (2020). Preliminary concerns with vaccine vectors. Mutagenesis 35, 359–360. doi: 10.1093/mutage/geaa020, PMID: 32785590

[ref14] Clear S. E. Dimmock J. A. Compton J. Jackson B. (2021). How do inoculation messages work? A two-study mixed-method investigation into inoculation mechanisms. Asian J. Commun. 31, 83–104. doi: 10.1080/01292986.2021.1888306

[ref15] Cohen J. (1988). Statistical power analysis for the behavioral sciences (2nd ed.). Hillsdale, NJ: Lawrence Erlbaum Associates, Publishers.

[ref16] Compton J. Ivanov B. (2012). Untangling threat during inoculation-conferred resistance to influence. Commun. Rep. 25, 1–13. doi: 10.1080/08934215.2012.661018

[ref17] Compton J. Jackson B. Dimmock J. A. (2016). Persuading others to avoid persuasion: inoculation theory and resistant health attitudes. Front. Psychol. 7:122. doi: 10.3389/fpsyg.2016.00122, PMID: 26903925 PMC4746429

[ref18] Compton J. Pfau M. (2004). Use of inoculation to foster resistance to credit card marketing targeting college students. J. Appl. Commun. Res. 32, 343–364. doi: 10.1080/0090988042000276014

[ref19] Compton J. A. Pfau M. (2005). Inoculation theory of resistance to influence at maturity: recent Progress in theory development and application and suggestions for future research. Ann. Int. Commun. Assoc. 29, 97–146. doi: 10.1080/23808985.2005.11679045

[ref20] Cook T. M. Roberts J. V. (2021). Impact of vaccination by priority group on UK deaths, hospital admissions and intensive care admissions from COVID-19. Anaesthesia 76, 608–616. doi: 10.1111/anae.15442, PMID: 33572007 PMC8013188

[ref21] Cornelis E. Cauberghe V. De Pelsmacker P. (2014). The inoculating effect of message sided-ness on adolescents’ binge drinking intention: the moderating role of issue involvement. J. Drug Issues 44, 254–268. doi: 10.1177/0022042613500053

[ref23] Dror A. A. Eisenbach N. Taiber S. Morozov N. G. Mizrachi M. Zigron A. (2020). Vaccine hesitancy: the next challenge in the fight against COVID-19. Eur. J. Epidemiol. 35, 775–779. doi: 10.1007/s10654-020-00671-y, PMID: 32785815 PMC8851308

[ref24] Ecker U. K. H. Sze B. K. N. Andreotta M. (2021). Corrections of political misinformation: no evidence for an effect of partisan worldview in a US convenience sample. Philos. Trans. Royal Soc B: Bio. Sci. 376:20200145. doi: 10.1098/rstb.2020.0145, PMID: 33612006 PMC7934973

[ref25] Eisend M. (2006). Two-sided advertising: a meta-analysis. Int. J. Res. Mark. 23, 187–198. doi: 10.1016/j.ijresmar.2005.11.001

[ref26] Ghebreyesus T. A. (2020). WHO director-General’s opening remarks at the media briefing on COVID-19. World Health Organization, 11. Available at: https://www.who.int/director-general/speeches/detail/who-director-general-s-opening-remarks-at-the-media-briefing-on-covid-19---11-march-2020 (Accessed March 11, 2020.)

[ref27] Government of Hong Kong Special Administrative Region (2021). Protect yourself and others get vaccinated. Available at: https://www.covidvaccine.gov.hk/en/dashboard (Accessed June 6, 2022).

[ref29] Horton R. (2020). Offline: managing the COVID-19 vaccine infodemic. Lancet 396:1474. doi: 10.1016/S0140-6736(20)32315-1, PMID: 33160553 PMC7834368

[ref30] Islam M. S. Sarkar T. Khan S. H. Mostofa Kamal A.-H. Hasan S. M. M. Kabir A. (2020). COVID-19–related infodemic and its impact on public health: a global social media analysis. Am. J. Trop. Med. Hygiene 103, 1621–1629. doi: 10.4269/ajtmh.20-0812, PMID: 32783794 PMC7543839

[ref31] Ivanov B. (2017). Inoculation theory applied in health and risk messaging. Oxford University Press.

[ref32] Ivanov B. Pfau M. Parker K. A. (2009a). The attitude base as a moderator of the effectiveness of inoculation strategy. Commun. Monogr. 76, 47–72. doi: 10.1080/03637750802682471

[ref33] Ivanov B. Pfau M. Parker K. A. (2009b). Can inoculation withstand multiple attacks?: an examination of the effectiveness of the inoculation strategy compared to the supportive and restoration strategies. Commun. Res. 36, 655–676. doi: 10.1177/0093650209338909

[ref34] Jin K.-X. (2020). Keeping people safe and informed about the coronavirus. Facebook. Available at: https://about.fb.com/news/2020/12/coronavirus/ (Accessed June 6, 2022).

[ref35] Jolley D. Douglas K. M. (2017). Prevention is better than cure: addressing anti-vaccine conspiracy theories. J. Appl. Soc. Psychol. 47, 459–469. doi: 10.1111/jasp.12453

[ref36] Lazarus J. V. Ratzan S. C. Palayew A. Gostin L. O. Larson H. J. Rabin K. (2021). A global survey of potential acceptance of a COVID-19 vaccine. Nat. Med. 27, 225–228. doi: 10.1038/s41591-020-1124-9, PMID: 33082575 PMC7573523

[ref37] Lee H. Cameron G. T. (2017). Utilizing audiovisual and gain-framed messages to attenuate psychological reactance toward weight management health messages. Health Commun. 32, 72–81. doi: 10.1080/10410236.2015.1099506, PMID: 27159448

[ref39] Leung K. Low Z. Sun F. (2021), Hong Kong may ease Covid-19 measures, but app rule draws business fears. South China Morning Post. Available at: https://www.scmp.com/news/hong-kong/health-environment/article/3121252/coronavirus-hong-kong-set-reveal-planned-changes (Accessed June 6, 2022).

[ref40] Lewandowsky S. Ecker U. K. H. Seifert C. M. Schwarz N. Cook J. (2012). Misinformation and its correction: continued influence and successful Debiasing. Psychol. Sci. Public Interest 13, 106–131. doi: 10.1177/1529100612451018 26173286

[ref41] Loomba S. de Figueiredo A. Piatek S. J. de Graaf K. Larson H. J. (2021). Measuring the impact of COVID-19 vaccine misinformation on vaccination intent in the UK and USA. Nat. Hum. Behav. 5, 337–348. doi: 10.1038/s41562-021-01056-1, PMID: 33547453

[ref42] Maertens R. Anseel F. van der Linden S. (2020). Combatting climate change misinformation: evidence for longevity of inoculation and consensus messaging effects. J. Environ. Psychol. 70:101455. doi: 10.1016/j.jenvp.2020.101455

[ref43] Marien S. Hooghe M. (2011). Does political trust matter? An empirical investigation into the relation between political trust and support for law compliance. Eur J Polit Res 50, 267–291. doi: 10.1111/j.1475-6765.2010.01930.x

[ref44] McGuire W. J. (1961). Resistance to persuasion conferred by active and passive prior refutation of the same and alternative counterarguments. J. Abnorm. Soc. Psychol. 63, 326–332. doi: 10.1037/h0048344

[ref45] McGuire W. J. (1964). “Inducing resistance to persuasion: some contemporary approaches,” in Advances in experimental social psychology. ed. Berkowitz L., vol. 1 (New York,NY: Academic Press), 191–229.

[ref46] Miller C. H. Ivanov B. Sims J. Compton J. Harrison K. J. Parker K. A. (2013). Boosting the potency of resistance: combining the motivational forces of inoculation and psychological reactance. Hum. Commun. Res. 39, 127–155. doi: 10.1111/j.1468-2958.2012.01438.x

[ref47] Min C. Shen F. Yu W. Chu Y. (2020). The relationship between government trust and preventive behaviors during the COVID-19 pandemic in China: exploring the roles of knowledge and negative emotion. Prev. Med. 141:106288. doi: 10.1016/j.ypmed.2020.106288, PMID: 33091414 PMC7571476

[ref48] Mukandavire Z. Nyabadza F. Malunguza N. J. Cuadros D. F. Shiri T. Musuka G. (2020). Quantifying early COVID-19 outbreak transmission in South Africa and exploring vaccine efficacy scenarios. PLoS One 15:e0236003. doi: 10.1371/journal.pone.0236003, PMID: 32706790 PMC7380646

[ref49] Murphy J. Vallières F. Bentall R. P. Shevlin M. McBride O. Hartman T. K. (2021). Psychological characteristics associated with COVID-19 vaccine hesitancy and resistance in Ireland and the United Kingdom. Nat. Commun. 12:29. doi: 10.1038/s41467-020-20226-9, PMID: 33397962 PMC7782692

[ref50] Nabi R. L. (2003). " feeling" resistance: exploring the role of emotionally evocative visuals in inducing inoculation. Media Psychol. 5, 199–223. doi: 10.1207/S1532785XMEP0502_4

[ref51] Nabi R. L. Moyer-Gusé E. Byrne S. (2007). All joking aside: a serious investigation into the persuasive effect of funny social issue messages. Commun. Monogr. 74, 29–54. doi: 10.1080/03637750701196896

[ref52] O’Keefe D. J. Nan X. (2012). The relative persuasiveness of gain- and loss-framed messages for promoting vaccination: a meta-analytic review. Health Commun. 27, 776–783. doi: 10.1080/10410236.2011.640974, PMID: 22292904

[ref53] Our World in Data (2021). Statistics and research coronavirus (COVID-19) vaccinations. Available at: https://ourworldindata.org/covid-vaccinations (Accessed June 6, 2022).

[ref54] Parker K. A. Ivanov B. Compton J. (2012). Inoculation’s efficacy with young adults’ risky behaviors: can inoculation confer cross-protection over related but untreated issues? Health Commun. 27, 223–233. doi: 10.1080/10410236.2011.575541, PMID: 21854225

[ref55] Pfau M. (1995). “Designing messages for behavioral inoculation” in Designing health messages: Approaches from communication theory and public health practice. eds. Maibach E. Parrott R. L. (Thousand Oaks, CA: Sage), 99–113.

[ref56] Pfau M. Ivanov B. Houston B. Haigh M. Sims J. Gilchrist E. (2005). Inoculation and mental processing: the instrumental role of associative networks in the process of resistance to counterattitudinal influence. Commun. Monogr. 72, 414–441. doi: 10.1080/03637750500322578

[ref58] Pfau M. Semmler S. M. Deatrick L. Mason A. Nisbett G. Lane L. (2009b). Nuances about the role and impact of affect in inoculation. Commun. Monogr. 76, 73–98. doi: 10.1080/03637750802378807

[ref59] Pfau M. Tusing K. J. Koerner A. F. Lee W. Godbold L. C. Penaloza L. J. (1997). Enriching the inoculation construct: the role of critical components in the process of resistance. Hum. Commun. Res. 24, 187–215. doi: 10.1111/j.1468-2958.1997.tb00413.x

[ref60] Pfau M. Tusing K. J. Lee W. Godbold L. C. Koerner A. Penaloza L. J. (2009a). Nuances in inoculation: the role of inoculation approach, ego-involvement, and message processing disposition in resistance. Commun. Q. 45, 461–481. doi: 10.1080/01463379709370077

[ref61] Pfau M. van Bockern S. Kang J. G. (1992). Use of inoculation to promote resistance to smoking initiation among adolescents. Commun. Monogr. 59, 213–230. doi: 10.1080/03637759209376266

[ref62] Randolph H. E. Barreiro L. B. (2020). Herd immunity: understanding COVID-19. Immunity 52, 737–741. doi: 10.1016/j.immuni.2020.04.012, PMID: 32433946 PMC7236739

[ref63] Reuters. (2021). Polls shows Hong Kong residents’ distrust of Chinese vaccines. Reuters. Available at: https://www.reuters.com/article/us-health-coronavirus-hongkong-idUSKBN29X10P (Accessed June 6, 2022).

[ref64] Richards A. S. Banas J. A. Magid Y. (2017). More on inoculating against reactance to persuasive health messages: the paradox of threat. Health Commun. 32, 890–902. doi: 10.1080/10410236.2016.1196410, PMID: 27435518

[ref65] Silvia P. J. (2006). Reactance and the dynamics of disagreement: multiple paths from threatened freedom to resistance to persuasion. Eur. J. Soc. Psychol. 36, 673–685. doi: 10.1002/ejsp.309

[ref66] Swire B. Ecker U. K. H. Lewandowsky S. (2017). The role of familiarity in correcting inaccurate information. J. Exp. Psychol. Learn. Mem. Cogn. 43, 1948–1961. doi: 10.1037/xlm0000422, PMID: 28504531

[ref67] Van Bavel J. J. Baicker K. Boggio P. S. Capraro V. Cichocka A. Cikara M. (2020). Using social and behavioural science to support COVID-19 pandemic response. Nat. Hum. Behav., 4, 460–471. doi: 10.1038/s41562-020-0884-z 32355299

[ref68] van der Linden S. Dixon G. Clarke C. Cook J. (2021). Inoculating against COVID-19 vaccine misinformation. EClinicalMedicine 33:100772. doi: 10.1016/j.eclinm.2021.100772, PMID: 33655205 PMC7908879

[ref69] van der Linden S. Roozenbeek J. Compton J. (2020). Inoculating against fake news about COVID-19. Front. Psychol. 11:566790. doi: 10.3389/fpsyg.2020.566790, PMID: 33192844 PMC7644779

[ref70] Vosoughi S. Roy D. Aral S. (2018). The spread of true and false news online. Science 359, 1146–1151. doi: 10.1126/science.aap9559 29590045

[ref71] Voysey M. Costa Clemens S. A. Madhi S. A. Weckx L. Y. Folegatti P. M. Aley P. K. (2021). Single dose administration, and the influence of the timing of the booster dose on immunogenicity and efficacy of ChAdOx1 nCoV-19 (AZD1222) vaccine. Lancet [Preprint]. https://papers.ssrn.com/abstract=3777268, 397, 881, 891, doi: 10.1016/S0140-6736(21)00432-3, PMID: 33617777 PMC7894131

[ref72] Walter N. Murphy S. T. (2018). How to unring the bell: a meta-analytic approach to correction of misinformation. Commun. Monogr. 85, 423–441. doi: 10.1080/03637751.2018.1467564

[ref470] Wang Y. McKee M. Torbica A. Stuckler D. (2019). Systematic literature review on the spread of health-related misinformation on social media. Soc. Sci. Med. 240:112552., PMID: 31561111 10.1016/j.socscimed.2019.112552 PMC7117034

[ref73] WHO (2022). Infodemic. Available at: https://www.who.int/health-topics/infodemic#tab=tab_1 (Accessed June 6, 2022).

[ref74] Wong N. C. H. (2014). Predictors of information seeking about the COVID-19 vaccine from parents and doctors among young college women. Commun. Q. 62, 75–96. doi: 10.1080/01463373.2013.860905

[ref75] Wong N. C. H. (2016). “Vaccinations are safe and effective”: inoculating positive HPV vaccine attitudes against antivaccination attack messages. Commun. Rep. 29, 127–138. doi: 10.1080/08934215.2015.1083599

[ref76] Wong M. C. S. Wong E. L. Y. Huang J. Cheung A. W. L. Law K. Chong M. K. C. (2021). Acceptance of the COVID-19 vaccine based on the health belief model: a population-based survey in Hong Kong. Vaccine 39, 1148–1156. doi: 10.1016/j.vaccine.2020.12.083, PMID: 33461834 PMC7832076

[ref77] Wood M. L. M. (2007). Rethinking the inoculation analogy: effects on subjects with differing preexisting attitudes. Hum. Commun. Res. 33, 357–378. doi: 10.1111/j.1468-2958.2007.00303.x

[ref79] Zaichkowsky J. L. (1985). Measuring the involvement construct. J. Consum. Res. 12, 341–352. doi: 10.1086/208520

[ref80] Zhu Q. Skoric M. Shen F. (2017). I shield myself from thee: selective avoidance on social media during political protests. Polit. Commun. 34, 112–131. doi: 10.1080/10584609.2016.1222471

