# Supplementary material for: Inoculation works and health advocacy backfires: Building resistance to COVID-19 vaccine misinformation in a low political trust context
Source: Front Psychol. 2022 Oct 25;13:976091. doi: 10.3389/fpsyg.2022.976091 (PMC9641202; doi:10.3389/fpsyg.2022.976091)
Supplement: Supplementary file 1 [file Data_Sheet_1.PDF]

Supplementary Material

1 Assumption tests for main variables

1.1 Normality

The normality of all the continuous variables (P1-P3 vaccine attitudes, P1-P3 vaccine intention, political trust, issue involvement, and age) was examined by the Kolmogorov-Smirnov and Shapiro-Wilk tests of normality in SPSS. All the Kolmogorov-Smirnov and Shapiro-Wilk statistics provided little evidence against the null hypothesis of normal distribution (for Kolmogorov-Smirnov tests, p-value = 0.56~0.20; for Shapiro-Wilk tests, p-value = 0.60 ~0.24). The statistics of skewness and kurtosis all fell into acceptable ranges (skewness = -0.658, 0.691; kurtosis = -0.552, 0.592).

1.2 Linearity

We constructed matrix scatter plots in SPSS graph builder. All the main variables had linear relationships with each other.

2 Assumption tests for Multivariate Analysis of Covariance (MANCOVA)

2.1 Variable types

Dependent variables (P3 vaccine attitudes, P3 vaccine intention, and Attitudinal threat) and covariates (P1 vaccine attitude) are continuous variables. The independent variable (experimental condition) is categorical.

2.2 Homogeneity of Variance

Levene's Test of Equality of Variance was used to examine whether the variance of dependent variables was equal across experimental conditions. All three the Levene's tests indicated equal variance between conditions (p-value = 0.17 ~ 0.94).

2.3 Homoscedasticity

Box's M Test was used to examine the equality of variance-covariance matrices. The test provided little evidence against the null hypothesis of multivariate homogeneity of variance (p = 0.086).

2.4 Homogeneity of regression slopes

The significance level of Wilks' lambda for the covariate cross-product (condition x P1 vaccine attitude) was 0.134, so the assumption of homogenous regression slopes stands.

2.5 Multicollinearity

Multicollinearity in MANCOVA is usually evaluated by inspecting the results of the residuals SSCP matrix. All the correlations between our main variables were below 0.8.

We also used linear regression in SPSS to produce the variance inflation factor (VIF) to spot any multicollinearity. All VIF factors fell into the range of 1.33~2.80 in Model 1 and Model 2, thus

we concluded that multicollinearity was not present in the data (see Table 1 for the VIF values in Model 1, and Table 2 for VIF values in Model 2).

Table 1
Multicollinearity diagnostics for multiple linear regression on P3 vaccine attitude (Model 1)

Variable	VIF
Gender	1.16
Age	1.16
Influenza vaccination history	1.15
Experimental Condition	1.17
Involvement	1.70
Political trust	1.16
Perceived threat	1.15
Counterarguing category	1.33
P1 vaccine attitude	1.73
P2 vaccine attitude	2.80

Table 2
Multicollinearity diagnostics for multiple linear regression on P3 vaccine intention (Model 2).

Variable	VIF
Gender	1.16
Age	1.15
Influenza vaccination history	1.17
Experimental Condition	1.15
Involvement	1.75
Political trust	1.35
Perceived threat	1.15
Counterarguing category	1.18
P1 vaccine attitude	1.69
P2 vaccine intention	2.20

2.7 Independent errors

The normality of distributed errors (residuals) was checked by crafting the Predicted Probability (P-P plot) in SPSS. No major deviations from the line were detected, which suggests that the distribution of errors was normal.

The Durbin-Watson test was performed to detect the presence of autocorrelation in the residuals. The Durbin-Watson estimates were 1.96 and 1.91 for Model 1 and Model 2, which fell between the critical cutoff of $1.5 < d < 2.5$. Thus, there was no linear autocorrelation in our data.

2.8 Independence of observations

Our experiment was conducted with randomized assignments of experimental conditions thus this assumption is met.
